# Effectiveness of Osteopathic Manipulative Treatment on Hemodynamic and Pulmonary Response in Coronary Artery Bypass Graft Patients: A Meta-Analysis

**DOI:** 10.7759/cureus.67968

**Published:** 2024-08-27

**Authors:** Courtney McGonegal, Shezore Bhatti, Jomar Carrasquillo, Mark A Potesta, James Kavulich, James Toldi

**Affiliations:** 1 Osteopathic Medicine, Lake Erie College of Osteopathic Medicine - Bradenton, Bradenton, USA; 2 Sports Medicine, Lincoln Memorial University DeBusk College of Osteopathic Medicine, Orange Park, USA

**Keywords:** omm, osteopathic manipulative medicine, omt, osteopathic manipulative treatment, cabg, coronary artery bypass graft, cardiac rehabilitation, cardiovascular disease, cardiology

## Abstract

In the realm of cardiovascular care, the quest for innovative and holistic approaches to enhance patient outcomes persists. This study analyzes osteopathic manipulative treatment (OMT) and its potential impact on pain intensity, length of hospitalization, respiratory function, and hemodynamic response in patients undergoing coronary artery bypass grafting (CABG). OMT, with its emphasis on physical manipulation of the body’s muscles and tissues, presents a potential treatment beyond the realms of conventional post-operative care. Google Scholar was used to identify four relevant articles for further review. RevMan 5.4 was utilized for meta-analytic evaluation in order to produce forest plots with associated standardized mean difference (SMD), confidence interval (CI), and heterogeneity (I²). Output from collection and analysis revealed statistically significant decreases in negatively viewed outcomes, including length of stay (SMD 0.39; 95% CI -0.02,-0.76; I² ≤ 0%) and pain control (SMD 1.67; 95% CI -1.34,-0.67; I² ≤ 94%). Parameters for respiratory function and hemodynamic response, including vital capacity (SMD 0.91; 95% CI 0.57,1.24; I² ≤ 96%) and maximal aerobic capacity (SMD 0.50; 95% CI 0.19, 0.82; I² ≤ 0%), showed a statistically significant increase. These findings suggest the incorporation of OMT as a viable adjunct for postoperative management in CABG patients, yielding favorable reductions in adverse outcomes such as length of hospitalization and pain. Moreover, it has demonstrated enhancement in maximal aerobic and vital capacity. This study suggests that the addition of osteopathic management to post-bypass standards can ultimately prevent certain morbidities associated with this specific patient population.

## Introduction and background

Background

Coronary artery bypass graft, also known as CABG, is the most common cardiovascular surgical procedure performed, with approximately 400,000 recorded each year [1]. This procedure is utilized to bypass an atherosclerotic obstruction within a major coronary vessel in an attempt to revascularize the ischemic myocardium. The bypass is constructed by harvesting a peripheral vein or arterial vessel from the patient, typically the left internal mammary artery or saphenous vein. The surgeon proceeds by forming an anastomosis both proximal and distal to the obstruction of the coronary artery with the harvested vessel. This anastomosis will permit arterial flow distal to the blockage, ensuring adequate oxygenation and nutrient delivery to the myocardium [1]. As with any surgical procedure, complications may arise. Following CABG surgery, a few of the most frequently reported cardiac and non-cardiac complications include myocardial infarction, mediastinitis and post-sternotomy pain, pleural effusions, decline in pulmonary function, postoperative ileus, bleeding, and stroke [2].

In osteopathic medicine, one of the five models of osteopathic care is the respiratory-circulatory model. This model highlights the essential nature of effective arterial supply, respiration, and venous and lymphatic drainage in the prevention and management of disease through the use of osteopathic manipulative treatment (OMT) [3]. Simultaneously, viscerosomatic responses in the T1-T4/5 thoracic region can originate from the cardiac system [3]. Myofascial release and soft tissue techniques localized to the thoracic spine, alongside rib raising and various lymphatic techniques, may benefit patients postoperatively to enhance hemodynamic stability and prevent complications. Each of these techniques focuses on eliminating restrictions to lymphatic flow and venous return, reflecting the potential for osteopathic therapies to aid patients in recovery from bypass surgery [3]. Discovering holistic, non-invasive approaches to enhance the cardiac rehabilitation process is of great interest. As such, the focus of this study is the efficacy of OMT therapy in post-CABG patients.

Aim

The aim of this meta-analysis is to determine the effectiveness of OMT in preventing or decreasing the number and duration of post-CABG complications while optimizing hemodynamic and pulmonary response.

Objectives

The objective of this meta-analysis is to determine the effect of OMT on each of the following primary end-points in post-CABG patients - hemodynamic response, pulmonary function, post-surgical pain, and length of stay. Based on these findings, should OMT be implemented as an adjunct to the post-CABG standard of care?

## Review

Methods

Google Scholar served as the primary source utilized in the process of screening for potential randomized controlled trials and case studies published regarding OMT and CABG procedures. The search terms included “osteopathic manipulative treatment CABG” with a customized date range of “1985-2023.” The first 50 pages of Google Scholar search results were screened on November 25, 2023, resulting in a total of 499 articles for review. Each researcher reviewed 10 pages of Google Scholar, with 50 total pages reviewed.

In order for the article to be included in this study, it must be written in English, contain human subjects, and be conducted on or after 1985. The study must also contain at least one of the following outcomes: hemodynamic response (maximal aerobic capacity), pulmonary function (vital capacity), postoperative length of stay, and pain intensity. Articles were then excluded if the outcomes were not reported quantitatively or as a mean and standard deviation.

**Figure 1:**
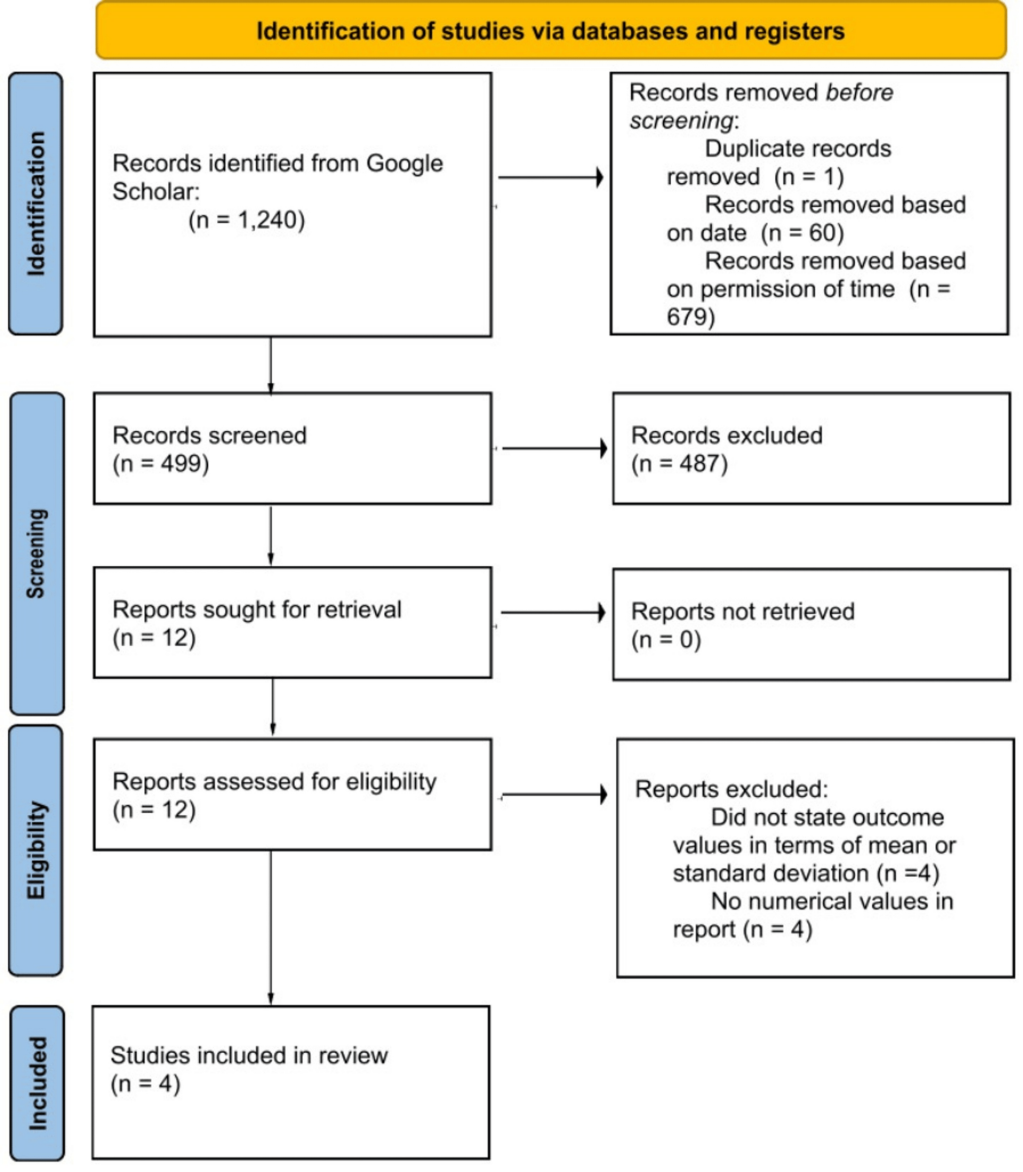
Preferred Reporting Items for Systematic Reviews and Meta-Analyses (PRISMA) literature review flow diagram

For the construction of the meta-analysis, 12 studies were reviewed and analyzed. Only four studies met all exclusion and inclusion criteria and thus were incorporated into the forest plots. The four studies that met our inclusion criteria were articles by Wieting et al., Racca et al., Roncada et al., and Ratajska et al. [4-7]. The remaining studies were excluded [8-12]. These studies were analyzed using RevMan 5.4, with a fixed-effects model and reporting of both standardized mean difference (SMD) and heterogeneity, I² [13]. A p-value less than 0.05 was accepted as statistically significant. Preferred Reporting Items for Systematic Reviews and Meta-Analyses (PRISMA) guidelines were followed in the literature screening process, as seen in Figure 1 [14].

Additionally, the NIH Study Quality Assessment Tool was utilized to determine bias associated with the included case-control studies [15]. The NIH Study Quality Assessment Tool is an assessment utilized in many meta-analyses to analyze the internal validity of each included study, ultimately permitting the evaluation of strengths and weaknesses associated with the research [15]. Two researchers were delegated the role of conducting the quality assessment. If a disagreement regarding answers to the provided questions occurred, a discussion was held between the two researchers until an agreed-upon decision was reached. An article was deemed “good” if there was less than or equal to one “no” in response to questions. An article was deemed “fair” if there were two to three “no” responses. Finally, an article was deemed “poor” in the quality assessment if there were more than three “no” responses.

Results

The literature search is summarized in Figure 1. Four studies met the inclusion criteria for this study with a total of 279 participants (n = 139 OMT, n = 140 control) [4-7]. Additionally, each of the articles was screened for bias. Three out of the four articles were deemed “good,” while one article, Ratajska et al., was determined “fair” with regard to quality assessment.

The outcomes measured in this meta-analysis were hemodynamic function via maximal aerobic capacity, pulmonary function via vital capacity, postoperative length-of-stay, and post-OMT pain scale. Table 1 details commonly reported outcomes, listed in order of highest to lowest frequency, along with references to each of the studies that reported the respective outcomes. As shown in Table 1, studies meeting inclusion criteria were marked. A comprehensive analysis summarizing all statistics and corresponding outcomes is found in Table 2.

**Table 1:** Literature review reported outcomes *Articles included in the meta-analysis [4-7]. CABG - coronary artery bypass graft; OMT - osteopathic manipulative treatment

Reported outcomes post-CABG OMT therapy	No. of studies	References
Decrease in pain intensity	7	*[4],*[5],*[6],*[7], [10], [11], [12]
Increase in pulmonary function	4	*[5],*[6],*[7], [9]
Decrease in length of stay	3	*[4],*[5], [8]
Increase in maximum aerobic capacity	2	*[5],*[6]
Increase in thoracic expansion	2	[9], [12]
Decreased time to postoperative bowel movement	1	*[4]
Increase in thoracic impedance	1	[8]
Increase in peripheral circulation	1	[8]
Increase in mixed venous oxygen saturation	1	[8]
Increase in cardiac index	1	[8]

**Table 2:** Summary of subtotal forest plot statistics I² - heterogeneity; OMT - osteopathic manipulative treatment; SMD - standardized mean difference Based on data obtained from Wieting et al. [4], Racca et al. [5], Roncada et al. [6], and Ratajska et al. [7].

Outcome measures	Control n	OMT n	SMD (95% CI)	p-value	I²
Vital capacity	82	82	0.91 (0.57, 1.24)	<0.00001	96%
Maximal aerobic capacity	82	82	0.50 (0.19, 0.82)	0.001	0%
Length of stay	57	58	-0.39 (-0.76, -0.02)	0.04	0%
Pain intensity	82	82	-1.00 (-1.34, -0.67)	<0.00001	95%

**Figure 2:**
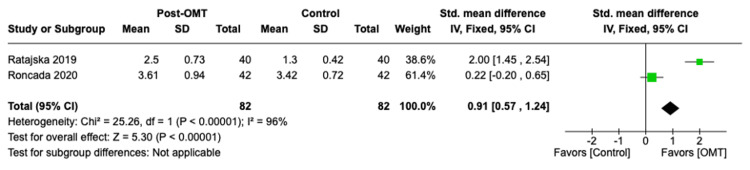
Vital capacity forest plot Roncada et al. [6]; Ratajska et al. [7].

**Figure 3:**
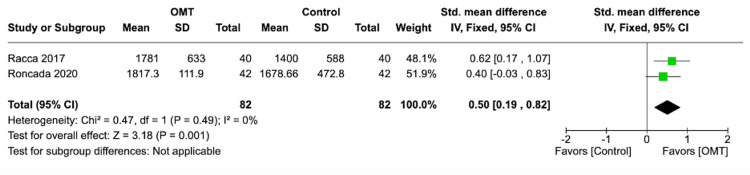
Maximal aerobic capacity forest plot Racca et al. [5]; Roncada et al. [6].

**Figure 4:**
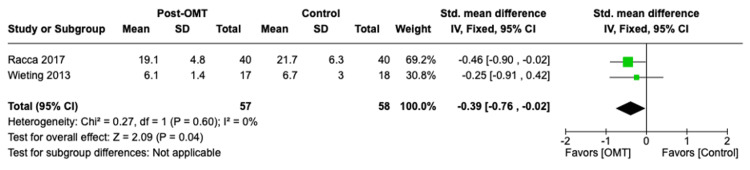
Length of stay forest plot Wieting et al. [4]; Racca et al. [5].

**Figure 5:**
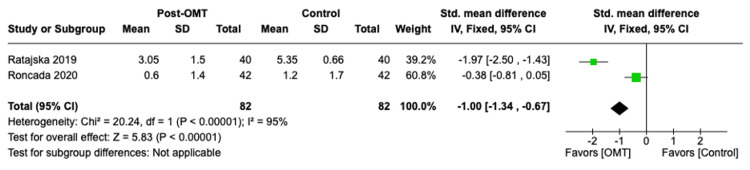
Pain intensity forest plot Roncada et al. [6]; Ratajska et al. [7].

Additionally, the forest plot results are displayed in Figures 2-5. Data analysis revealed a decrease in average pain intensity in OMT patients post sternotomy for CABG procedure compared to controls, with p < 0.00001 and high heterogeneity, I² ≤ 95%. The SMD was calculated to be -1.00 with a 95% CI of -1.34, -0.67. In the “length of stay” outcome, the calculated standardized mean was -0.39 with a 95% CI of -0.76, -0.02. The determined p-value was <0.04 with a high heterogeneity, I² ≤ 0%. The forest plot for “maximal aerobic capacity,” as shown in Figure 3, displayed an SMD of 0.50 with a 95% CI of 0.19, 0.82. The reported p-value was <0.001 with a low heterogeneity I² ≤ 0%. Lastly, the length of stay SMD was calculated to be 0.39 with a 95% CI of 0.02, 0.76. The p-value measured <0.04 and I² of 0%. The outcomes from individual studies and the quality analysis are displayed in Table 3 and Table 4, respectively.

**Table 3:** Outcome data from individual studies L - liter; mL - milliliter; OMT - osteopathic manipulative treatment; SD - standard deviation

	Studies	Post-OMT, mean ± SD	Controls, mean ± SD
3A: Vital capacity (L)	Roncada et al. [6]	3.61 ± 0.94	3.42 ± 0.72
	Ratajska et al. [7]	2.50 ± 0.73	1.30 ± 0.42
3B: Maximal aerobic capacity (mL)	Racca et al. [5]	1,781 ± 633	1,400 ± 588
	Roncada et al. [6]	1,817.3 ± 111.9	1,678.66 ± 472.8
3C: Length of stay (days)	Racca et al. [5]	19.1 ± 4.8	21.7 ± 6.3
	Wieting et al. [4]	6.1 ± 1.4	6.7 ± 3.0
3D: Pain intensity (visual analog scale)	Roncada et al. [6]	0.6 ± 1.4	1.2 ± 1.7
	Ratajska et al. [7]	3.05 ± 1.50	5.35 ± 0.66

**Table 4:** Quality assessment for case-control studies F - fair; N - no; NA - not applicable; G - good; Y - yes Based on the bias assessment tool [15].

Study	Case total (n)	Control total (n)	Bias	1	2	3	4	5	6	7	8	9	10	11	12
Racca et al., 2017 [5]	40	40	G	Y	Y	Y	Y	Y	Y	NA	Y	Y	Y	Y	Y
Ratajska et al., 2019 [7]	40	40	F	Y	Y	Y	Y	N	Y	NA	Y	Y	Y	N	Y
Roncada et al., 2020 [6]	42	42	G	Y	Y	Y	Y	Y	Y	NA	Y	Y	Y	Y	Y
Wieting et al., 2013 [4]	17	18	G	Y	Y	Y	Y	Y	Y	NA	Y	Y	Y	Y	Y

Discussion

CABG is highly utilized throughout the United States in the management of significant myocardial ischemia. Due to the substantial number of complications that may arise from this procedure, determining alternative ways to minimize and prevent postoperative complications remains a high priority. Multiple research articles have been published regarding the use of OMT following CABG procedures. The most commonly reported outcomes following the use of OMT in this specific patient population include a decrease in both pain intensity and length of hospitalization, with an enhancement in pulmonary function and hemodynamic response, as reported in Table 1. The aim of this study was to perform a meta-analysis on the effectiveness of OMT in decreasing and preventing post-CABG complications.

In the length-of-stay and maximal aerobic capacity outcomes, the OMT group demonstrated a significantly shorter hospitalization and increased peripheral circulation, respectively. Additionally, the I² values, as reported in Table 2 and Figures 3-4, indicated low heterogeneity, suggesting these studies demonstrated minimal variation with high generalizability to the greater post-CABG patient population. With an I² value of 0 in both forest plots, any variability in data would be attributed to sampling error in the studies. In the length-of-stay outcome, the pooled SMD was calculated to be -0.39, with a 95% CI of -0.76,-0.02. This indicates that the addition of post-OMT treatment is associated with shorter hospitalization compared to the standard of care alone. The pooled SMD reported in the maximal aerobic capacity outcome was 0.50, with a 95% CI of 0.19,0.82. The positive reflection of the SMD observed in the corresponding forest plot suggests that OMT aids in increasing maximal aerobic capacity in post-CABG patients. These hemodynamic responses to OMT techniques are a reflection of their ability to balance the response of the sympathetic and parasympathetic nervous systems alongside enhancing the motion of the osteopathic diaphragms [5]. Decreasing the sympathetic nervous system response via osteopathic techniques leads to decreased vasoconstrictive properties, ultimately enhancing oxygen delivery to vital tissues and organs. Improving aerobic respiration increases adenosine triphosphate production, thereby accelerating the pathologic process of healing and length of stay [16]. Both confidence intervals reported in length of stay and maximal aerobic capacity do not include 0, indicating statistical significance in addition to the p-values being <0.05.

As represented in Figure 2 and Figure 5, both pain intensity and vital capacity measurements were significantly lower in the OMT group compared to the control group, with p < 0.05. This indicates OMT was an effective intervention in decreasing postsurgical pain and increasing vital capacity following CABG procedures in the studies analyzed. The I² value was 94% in the “pain intensity” outcome and 96% in the “vital capacity” outcome, indicating a high heterogeneity between the included studies. Additionally, the pooled SMD of -1.00 with a 95% CI of -1.34, -0.67 for the pain intensity outcome supports the implementation of OMT in post-CABG patients with a statistically significant decrease in reported postsurgical pain. The calculated SMD for vital capacity was 0.91 with a 95% CI of 0.57, 1.24. This suggests that post-CABG OMT significantly increases measured vital capacity and pulmonary function post-surgically within the included studies. The ability of OMT to decrease post-surgical pain is likely associated with improving pulmonary function. The combination of decreasing pain intensity upon inspiration with increasing chest wall expansion via treatment of thoracic somatic dysfunctions through OMT likely contributes to this finding [6]. Decreasing pain intensity ultimately permits the patient to adequately inspire and expire, a physiologic function that is crucial in preventing the development of postoperative atelectasis, pleural effusion, and pneumonia. Additionally, decreasing postoperative pain has been associated with improved psychiatric outcomes. Cardiac pathologies and dysfunctions have been highly associated with acute-onset depression and anxiety, specifically in the post-CABG population [17]. Focusing on pain management through the simultaneous use of osteopathic therapy and standard-of-care demonstrates a profound effect on post-bypass morbidity and mortality. The I² values reported for these two outcomes differ quantitatively, suggesting more variability between studies. One potential source for the greater degree of heterogeneity is the subjective reporting of pain as a numerical value. This subjectivity may greatly affect the reported data between patients. Additionally, differing OMT techniques, the variation in time-of-initiation of OMT post-surgically, and the duration for which each technique was applied in each study are factors that may contribute to a high variation in data. The relatively small sizes of the included studies are an additional contributing factor to the low homogeneity.

A limitation of this meta-analysis is the limited number of research studies available for inclusion. This greatly affected sample sizes and, therefore, the power and heterogeneity of the study. Variations in heterogeneity among the studies may arise from a multitude of factors which can be attributed to methodological differences and/or population variability. As more randomized controlled trials are published regarding this topic, expansion of the meta-analysis can occur.

Overall, this meta-analysis has determined that OMT is statistically significant in its effect on enhancing maximal aerobic capacity and vital capacity measures while decreasing postsurgical pain and length-of-stay compared to the standard of care in patients undergoing CABG procedures. As more studies are published on this topic, future studies detailing proper initiation times post-surgically, duration, and separation of techniques associated with each of these findings may be more widely available for analysis and inclusion. This will further strengthen the association between the addition of OMT in post-CABG care and the effects observed on hemodynamic/pulmonary responses and post-surgical complications.

## Conclusions

The aim of this meta-analysis was to determine the effectiveness of OMT in preventing and decreasing the duration of post-CABG procedure complications while optimizing both hemodynamic stability and pulmonary function. This meta-analysis determined that OMT has a clinically significant effect on maximizing peripheral oxygenation and pulmonary function via increased vital capacity and decreasing reported pain intensity and length-of-stay post-surgically following CABG procedures. The hope, with the aid of future published data on this topic, is to navigate through the relationship between osteopathic principles and cardiac rehabilitation, highlighting the synergy between OMT and CABG and its effect on enhancing recovery with a deeper understanding of holistic patient care.
